# Integrating the DSM-5 Cultural Formulation Interview in the child mental health assessment in primary care

**DOI:** 10.1192/j.eurpsy.2026.10231

**Published:** 2026-07-31

**Authors:** K. Viksten Assel, J. Åhlén, S. Bäärnhielm, C. Dalman

**Affiliations:** 1 Department of Global Public Health; 2 Department of Clinical Neuroscience, Karolinska Institutet, Stockholm, Sweden

## Abstract

**Introduction:**

The DSM-5 Cultural Formulation Interview (CFI) is a tool meant to strengthen mental health assessments’ sociocultural sensitivity. While a growing number of studies have demonstrated the value of conducting CFI with adult patients, there is limited knowledge as to the value of using the tool in child mental health assessments.

**Objectives:**

In this study, we evaluated the standard child mental assessment in primary care when complemented with parental interviews with the CFI.

**Methods:**

We used both quantitative and qualitative methods. The effect of the CFI was aimed to be investigated in a quasi-experimental trial including children who had sought care at four primary care based mental health services in Region Stockholm, Sweden. We compared standard assessment to standard assessment enhanced with the CFI. Web questionnaires completed by parents were used to assess treatment credibility, working alliance, mental problems and health-related quality of life. Of the 248 participants required, only 57 had been recruited within 18 months, which led to a premature termination of the study. Qualitative in-depth interviews were conducted with ten parents and five clinicians about their experience of the CFI.

**Results:**

Findings showed no differences in treatment credibility and working alliance between groups but suggested that children allocated to standard assessment enhanced with CFI had significantly lower level of anxiety and depressive symptoms, and higher levels of health-related quality of life. These results should be treated with caution, due to the limited number of children included, and limited pre-treatment assessment. In the qualitative interviews, parents described having been able to share their story, and that the questions during the assessment with the CFI were relevant. However, parents emphasized the importance for the CFI to be used flexibly. Clinicians described an improved understanding of parents’ perspective and of the child’s sociocultural context. However, the CFI questions on cultural identity were described as challenging to ask, and some clinicians found the information produced from the CFI to be too extensive for the primary care context.

**Conclusions:**

While indicating the value of using the CFI in child mental health assessments, our findings highlight the need for a flexible, contextualized use of the tool, and for further refinement of the questions in the CFI.

**Disclosure of Interest:**

None Declared

